# A DedA Family Membrane Protein in Indium Extrusion in *Rhodanobacter* sp. B2A1Ga4

**DOI:** 10.3389/fmicb.2021.772127

**Published:** 2021-11-26

**Authors:** Joana B. Caldeira, Ana Paula Chung, Ana Paula Piedade, Paula V. Morais, Rita Branco

**Affiliations:** ^1^University of Coimbra, Centre for Mechanical Engineering, Materials and Processes, Department of Life Sciences, Coimbra, Portugal; ^2^University of Coimbra, Centre for Mechanical Engineering, Materials and Processes, Department of Mechanical Engineering, Coimbra, Portugal

**Keywords:** DedA family protein, indium resistance, gene mutation, indium bioaccumulation, gene complementation

## Abstract

Indium (In) is a critical metal widely used in electronic equipment, and the supply of this precious metal is a major challenge for sustainable development. The use of microorganisms for the recovery of this critical high-tech element has been considered an excellent eco-friendly strategy. The *Rhodanobacter* sp. B2A1Ga4 strain, highly resistant to In, was studied in order to disclose the bacterial mechanisms closely linked to the ability to cope with this metal. The mutation of the gene encoding for a DedA protein homolog, YqaA, affected drastically the In resistance and the cellular metabolic activity of strain *Rhodanobacter* sp. B2A1Ga4 in presence of this metal. This indicates that this protein plays an important role in its In resistance phenotype. The negative impact of In might be related to the high accumulation of the metal into the mutant cells showing In concentration up to approximately 4-fold higher than the native strain. In addition, the expression of the *yqaA* gene in this mutant reverted the bacterial phenotype with a significant decrease of In accumulation levels into the cells and an increase of In resistance. Membrane potential measurements showed similar values for native and mutant cells, suggesting that there was no loss of proton-motive force in the mutant cells. The results from this study suggest a potential role of this DedA family protein as a membrane transporter involved in the In efflux process. The mutant strain also has the potential to be used as a biotool in bioaccumulation strategies, for the recovery of In in biomining activities.

## Introduction

Indium (In) is quite rare in nature and is found as a trace element in the earth’s crust (50–200 ppm); however, it is an important metal supporting modern communication and electronic industry (Alfantazi and Moskalyk, 2003). In is increasingly used as In arsenide, In tin oxide, and gallium (Ga) In arsenide in a wide collection of electronic products including mobile phones, light-emitting diodes for displays, light sources and detectors, microcircuits, lasers, and bioimaging agents (Nguyen et al., 2020). With high demand, scarcity, and lack of feasible substitute, In is considered a critical metal, being included in the critical raw materials list of various agencies, including the United Nations Environment Programme, the European Commission, the US National Academy of Sciences, and the US Department of Energy (U.S. Department of Energy, 2011; European Commission, 2020). In nature, In is often found with other metals, Zn, Fe, Cu, Pb, and Sn, and is extracted as a by-product of mining operations mainly from Zn extraction (Schwarz-Schampera, 2014; U.S. Geological Survey, 2019). The huge demand for In has resulted in an increase in mining activities and fostered an interest in alternative In secondary sources such as mine tailings and end-of-life electronic equipment (e-waste). Electronic devices comprise valuable elements including In, which can be present as 100–400 ppm in liquid crystal displays (Rocchetti et al., 2015). Thus, e-waste has been considered a potential source of critical metals (Işıldar et al., 2019). In addition, the extensive mining activities and the manufacturing of the In-based thin films used in many electronic devices often involve a significant amount of water that is discharged to sewers releasing soluble toxic metal species (e.g., GaIII, InIII, arsenite (AsIII), and arsenate) (Fashola et al., 2016) and, consequently, the widespread environmental contamination. These metal-contaminated environments are often colonized by microbial communities well adapted to these tough conditions. Several metal resistance mechanisms are known in microorganisms, such as the change of the metal redox state, metal cell impermeability, secretion of metal chelating agents to the environment, metal sorption on the microbial surface, and metal efflux (Srivastava and Kowshik, 2013).

In has no known biological role, and very few studies of In–microbial interactions have been reported. *Pseudomonas fluorescens* was able to detoxify In by precipitation in an insoluble complex with phosphate (Anderson and Appanna, 1993). Two *Serratia fonticola* strains responded to the oxidative stress induced by In exposure through activation of an additional superoxide dismutase enzyme (Caldeira et al., 2020). Recently, *Rhodanobacter* sp. B2A1Ga4 strain isolated from mine sediments showed high resistance to Ga, aluminum (Al), and In (Caldeira et al., 2021). Moreover, this late study recognized the importance of iron uptake in the control of oxidative stress, particularly induced by In. Although there is a shortage of information related to bacterial transport systems for In, the literature reports that this metal is naturally accumulated by Gram-positive and Gram-negative bacteria (Ardehali and Mohammad, 1993). In uptake was successfully achieved at room temperature and over a pH range of 2.4–3.9 by the Gram-negative bacterium *Shewanella algae* (Ogi et al., 2012). In general, to avoid accumulation of metals into the cells, bacteria can contain transporters belonging to several families such as ABC (ATP-binding cassette), SMR (small multidrug resistance), MFS (major facilitator superfamily), MATE (multiple antibiotic and toxin extrusion), and RND (resistance-nodulation-division) (Nikaido, 2011).

Conventional technologies have been used for In recovery from liquid processes and waste streams, but these conventional methodologies show many disadvantages, such as high requirements for reagents and energy, high capital and operational costs, generation of toxic waste products, and low recovery yield from low In concentration streams (Nicomel et al., 2020).

Therefore, alternative methodologies to recover critical metals as In have been emphasized in the last few years. In recovery can start with metal leaching and subsequent In concentration processes such as accumulation inside the cells (bioaccumulation) or metal binding on the cellular surface (biosorption). The bioaccumulation of In has not been widely explored for the recovery of this metal, although it is an eco-friendly process with a great potential to be an alternative to conventional strategies. Several biobased accumulators have been proposed for the removal and recovery of different metals as arsenic, tungsten, nickel, cobalt, cadmium, copper, uranium, and mercurial species (Sousa et al., 2015; Diep et al., 2018; Coimbra et al., 2019).

In the present work, an In-sensitive mutant of strain *Rhodanobacter* sp. B2A1Ga4 obtained by random mutagenesis was studied to correlate the mutant higher In susceptibility with an increase of In amount into the cells. The mutated gene was identified as encoding for a DedA family protein, and its potential role as an In transporter protein involved in In efflux was explored. The complementation of mutant with a plasmid expressing the DedA family protein reverted the In strain phenotype, indicating that the transporter is relevant to extrude In from cells.

## Materials and Methods

### Bacterial Strains, Media, and Growth Conditions

The bacterial strains and plasmids used in this work are listed in Table 1. The highly In resistant strain *Rhodanobacter* sp. B2A1Ga4 (Caldeira et al., 2021) was isolated from Panasqueira mine, Portugal. The growth of *Rhodanobacter* strains was performed using Reasoner’s 2A broth medium (R2Ab), containing the following per liter: 0.5 g yeast extract, 0.5 g proteose peptone, 0.5 g casein, 0.5 g glucose, 0.5 g soluble starch, 0.3 g K_2_HPO_4_, 0.024 g MgSO_4_, and 0.3 g sodium pyruvate. The growth of *Escherichia coli*, for plasmid construction, was performed in Luria-Bertani medium (LB), containing the following per liter: 10 g tryptone, 5 g yeast extract, and 5 g NaCl. Solid media were prepared with addition of 15 g per liter of agar to the liquid media. The growth of *Rhodanobacter* strains was evaluated by measuring the optical density at 600 nm (OD_600_) after different incubation times at 25°C. Metal stock solutions were prepared at concentrations of 0.5 M indium(III) chloride (InCl_3_) (Acros Organics), 0.2 M Ga(III) nitrate (GaN_3_O_9_) (Alfa Aesar), 0.2 M aluminum chloride (AlCl_3_) (Acros Organics), 0.1 M nickel(II) chloride (NiCl_2_) (Merck), 0.2 M neodymium(III) chloride (NdCl_3_) (Alfa Aesar), 0.05 M yttrium(III) chloride (YCl_3_) (Sigma), 0.05 M scandium(III) chloride (ScCl_3_) (Alfa Aesar), 1 M sodium arsenate dibasic (Na_2_AsO_4_) (Sigma), 0.2 M lanthanum(III) chloride (LaCl_3_) (Alfa Aesar), 1 M cooper(II) chloride (CuCl_2_) (Merck), 1 M zinc chloride (ZnCl_2_) (Sigma), 0.25 M sodium tellurite (Na_2_TeO_3_) (Sigma), and 1 M sodium chromate (Na_2_CrO_4_) (Sigma) and were sterilized by filtration.

**TABLE 1:** Bacterial strains and plasmids used in this work.

Strains or plasmids	Relevant characteristic(s)	Source or references
Strains		
*Rhodanobacter* sp. B2A1Ga4	Wild type, In resistant	Caldeira et al., 2021
*E. coli* S17-1	Mobilization host, recA pro hsdR RP4-2-Tc:Mu-Km:Tn7 integrated into the chromosome	DSMZ
Mutant B2	B2A1Ga4 strain with *yqaA* gene interrupted by Tn*5* transposon	This study
B2_p	Mutant B2 complemented with pBBR1MCS-5 empty plasmid	This study
B2_p*yqaA*	Mutant B2 complemented with *yqaA* gene cloned into pBBR1MCS-5 plasmid	This study
Plasmids		
pSUP5011	Tn*5*-based transposon	DSMZ
pBBR1MCS-5	Gmr; oripBBR1MCS Mob lacZa, broad-host-range cloning and expression vector	Kovach et al., 1995
p*yqaA*	pBBR1MCS-5 carrying the *yqaA* gene of B2A1Ga4 strain	This study

### Transposon Mutagenesis and Screening

An In susceptible B2A1Ga4 strain was obtained by random mutagenesis, by mobilization of the suicide plasmid pSUP5011 from the donor strain *E. coli* S17-1 to the recipient strain B2A1Ga4, using the filter plating methodology as described previously (Caldeira et al., 2021). The transconjugants grown on R2A plates supplemented with 100 μg/mL kanamycin and 50 μg/mL phosphomycin at 25°C were replicated to R2A media with 1 mM In. The clones unable to grow in the presence of this concentration of In for 5 days were recovered and subjected to further analyses.

### Inverse Polymerase Chain Reaction

The interrupted gene in mutant strain was analyzed by inverse polymerase chain reaction (IPCR) as described previously (Caldeira et al., 2021). Briefly, 1 μg of total bacterial DNA was digested with different restriction enzymes. Ligation reactions were prepared using 0.5 μg of digested DNA with 10 U of enzyme T4 ligase (ThermoFisher Scientific) in a final volume of 50 μL and incubated overnight at 16°C. After ligation and purification, PCR mixtures were prepared in a final volume of 50 μL containing 0.5 μg ligated DNA, 0.04 U/μL Platinum™ Taq DNA polymerase (Invitrogen, ThermoFisher Scientific), PCR buffer (1×), 1.5 mM MgCl_2_, 2% KB extender, 0.3 mM nucleoside triphosphates (dNTPs), and 0.4 μM of each primer (forward tn5_1775f: CCT TGC GCA GCT GTG CTC GAC GTT GTC ACT and reverse IR2: CGG GAT CCT CAC ATG GAA GTC A). DNA amplification was performed for 4 min at 94°C, followed by 30 cycles of 45 s at 94°C, 45 s at 60°C and 6 min at 68°C. The IPCR products were visualized on 1% agarose gel; the DNA bands were removed, purified using the E.Z.N.A. ^®^ Gel Extraction Kit (Omega Bio-Tek), and sequenced (STAB VIDA, Portugal). The sequences were submitted to BLAST in order to identify the interrupted gene by the transposon insertion.

### Sequence Alignment and Analysis

Alignment of YqaA protein sequence of *Rhodanobacter* sp. B2A1Ga4 with representative sequences of the DedA protein family was generated by ClustalW (Thompson et al., 1994). The selection of the representative DedA proteins from the NCBI database was based on previous studies that gave some insights about the possible roles, or evolutionary relationships among the DedA domain containing proteins (Supplementary Table 1). Protein location and secondary structure predictions were obtained by using the server PSORTb v 3.0.3 (Yu et al., 2010) and both programs TMHMM server 2.0 and CCTOP (Constrained Consensus Topology Prediction) (Krogh et al., 2001; Dobson et al., 2015).

### Minimum Inhibitory Concentration Assays and Bacterial Growths

The minimum inhibitory concentrations (MICs) for In were determined using the standard broth microdilution method in R2Ab medium for native and mutant B2 strains, and in R2Ab medium supplemented with gentamicin for complemented strains, B2_p and B2_p*yqaA*. Bacterial growths were analyzed after 48 h of incubation at 25°C. MIC values were the lowest concentration of In that inhibited a visible growth of the microorganism.

The resistance of the mutant strain to In and to other metals from the same group of the periodic table (Ga and Al) was compared with the wild strain, through the evaluation of their bacterial growth curves in presence of these metals. Both strains were grown in R2Ab medium in four different conditions: control (without metal), 0.4 mM Ga, 0.4 mM Al, and 0.2 mM In. Growths were performed at 25°C, 140 revolutions/min (rpm), and evaluated measuring the OD_600_ for 24 h.

The ability of B2A1Ga4 and mutant B2 strains to grow in presence of other metals, antibiotics, SDS, and at different temperatures was also evaluated to assess the potential effects of the gene mutation under different conditions. The resistance of both strains to metals and SDS at different concentrations was evaluated in R2Ab medium, in triplicate, measuring the OD_600_ after 48-h incubation at 25°C. The antibiotic resistance profiles were evaluated using the standardized disc susceptibility testing method (Jorgensen and Turnidge, 2007), and results were obtained after 48 h of incubation at 25°C. Finally, the temperature assays were performed by growing both strains in R2A medium at different temperatures (20, 25, 30, 37, 40°C) for 5 days.

The complemented strains B2_p*yqaA* and B2_p were grown in R2Ab medium supplemented with gentamicin (15 μg/mL) at 25°C, 140 rpm, without or with different concentrations of In (0.1 and 0.2 mM In), and In resistance was evaluated measuring OD600 at two times of incubation, 16 and 24 h, which correspond to the end of exponential growth phase and to the stationary growth phase, respectively.

### Test of Cellular Metabolic Activity

MTT assay (3-(4,5-dimethylthiazol-2-yl)-2,5-diphenyl tetrazolium bromide assay) was used to evaluate the cellular metabolic activity based on the protocol previously described (Wang et al., 2010). Briefly, samples from growths in the presence and absence of In were collected at two different times. Samples were centrifuged and washed twice, and the pellets were resuspended in 1 mL R2Ab medium. The cell suspensions were diluted to an OD_600_ of 0.2 with the medium. Reactions were prepared with 200 μL of the diluted cell suspensions and 20 μL of MTT stock solution (5 g/mL) and were incubated with the cap open at 25°C for 1 h. After the incubation time, the mixtures were centrifuged at 10,000*g* for 2 min, and the supernatant was discarded. The pellets (crystals) were dissolved in 2.5 mL of dimethyl sulfoxide (DMSO), and 1 h later, the samples were quantified spectrophotometrically at 550 nm.

### Membrane Potential

The membrane potential was measured according to a described protocol (Sikdar et al., 2013). Strains were grown in presence of 0.2 mM In and without metal for 10 h at 25°C and 140 rpm. Samples of 2 mL of growths with OD_600_ of approximately 0.8 were collected (the sample volumes of growths with lower OD_600_ were suitably adjusted). The pellets were resuspended with the working solution JC-1: 16 μL DMSO, 4 μL JC-1 stock solution (5 mg/mL), and 1 mL of permeabilization buffer (10 mM Tris, pH 7.5, 1 mM EDTA, 10 mM glucose). After 20-min incubation at 30°C (in the dark), the fluorescence was read: green fluorescence (λ_*em*_ = 530 nm and λ_*ex*_ = 485 nm) and red fluorescence (λ_*em*_ = 595 nm and λ_*ex*_ = 485 nm). Control experiments were performed with cells previously treated with 100 μM (3-chlorophenyl) hydrazonomalononitrile (CCCP) for 30 min. The results were obtained by the ratio fluorescence (red/green).

### Indium Quantification

To quantify the total of In accumulated by cells, strains were grown in R2Ab medium supplemented with 0.1 mM In at 25°C with 140 rpm, and cells were collected at different growth times. Samples were centrifuged at 4,000 rpm for 20 min at 4°C, the cellular pellets were washed twice with cold phosphate-buffered saline solution (containing the following per liter: 8 g NaCl, 0.2 g KCl, 1.44 g Na_2_HPO_4_, 0.24 g KH_2_PO_4_, pH 7.4), and the final cellular pellets were lysed by acid treatment (5% HNO_3_), heated at 50°C for 1 h, and then centrifuged at 13,000 rpm for 10 min. The In amount in the intracellular supernatants was quantified by inductively coupled plasma mass spectrometry (ICP-MS). Pellets were neutralized with NaOH 0.5 M and then used to quantify the total protein concentration by Bradford method (Bradford, 1976).

In addition, in specific assays, In was also quantified to differentiate the intracellular and extracellular In accumulation. Native and mutant strains were grown in presence of 0.1 mM In; samples were collected, centrifuged, and washed as previously described. The cellular pellets were resuspended with a solution of 20 mM EDTA and incubated for 15 min with 100-rpm agitation (Hudek et al., 2009). The samples were centrifuged, and the supernatants were used for quantification of In by ICP-MS, which correspond to the cellular surface metal binding fraction (biosorption fraction). The pellet was lysed by acid treatment (5% HNO_3_), heated at 50°C for 1 h, and then centrifuged at 13,000 rpm for 10 min. These supernatants were quantified by ICP-MS that correspond to intracellular metal fraction.

### Complementation of Mutant B2 With Plasmids

The mutant B2 was complemented with the empty vector pBBR1MCS-5, which served as a control, and with this same vector carrying the *yqaA* gene of B2A1Ga4 strain.

The *yqaA* gene was amplified from strain B2A1Ga4. PCR reaction consisted of a mixture of 2 ng B2A1Ga4 DNA, 0.04 U/μL Platinum™ Taq DNA polymerase (Invitrogen, ThermoFisher Scientific), PCR buffer (1×), 1.5 mM MgCl_2_, 0.3 mM dNTPs, 0.4 μM of specific forward and reverse primers comprising the enzyme restriction sequences for *Hin*dIII and *Xba*I, respectively, and sterile ultrapure water until the final volume of 50 μL. The PCR program was 4 min at 94°C, followed by 30 cycles of 1 min at 94°C, 1 min at 60°C, and 1 min at 72°C.

To clone the amplified gene into the plasmid pBBR1MCS-5, the PCR product and the plasmid were digested with the restriction enzymes *Hin*dIII and *Xba*I (Takara) for 2 h at 37°C. After purification of the restriction reactions, the gene was ligated to the digested vector through T4 ligase enzyme (ThermoFisher) to obtain p*yqaA* plasmid. The ligation reaction was purified with the E.Z.N.A. ^®^ Gel Extraction Kit (Omega Bio-Tek) and transformed into competent *E. coli* S17-1 cells. A clone from plates of LB agar with 100 μg/mL ampicillin and 15 μg/mL gentamicin was selected to colony-PCR reaction, and the PCR product was used to confirm the correct construction by sequencing (STAB VIDA, Portugal).

The p*yqaA* construction and the empty plasmid were mobilized into the B2 mutant by biparental separated conjugations (del Campo et al., 2012). The conjugated clones were obtained on plates of R2A supplemented with 100 μg/mL kanamycin and 15 μg/mL gentamicin incubated at 25°C for 3 days. A clone designated B2_p and a clone designated B2_p*yqaA* were selected for further assays.

### Scanning Electron Microscopy

A scanning electron microscopy (SEM) equipment, Vega3 from Tescan GmbH, was used to observe possible morphological surface cell changes in strains when exposed to In. Native and mutant cells were grown in R2Ab medium in presence of 0.1 mM In. After 24 h of incubation, the OD600 was measured, and bacterial suspensions were prepared to a final OD600 of approximately 0.3; 10 μL of these bacterial suspensions was placed on sterilized surfaces, fixated with 2.5% (vol/vol) of glutaraldehyde, and dehydrated by a grade of ethanol incubations [70, 80, 90, 95, and 100% (vol/vol)]. The bacterial suspension was shaken for 20 min in the glutaraldehyde solution and in each ethanol solution (Kaláb et al., 2008). Prior to observations, the samples were sputtered coated with a thin film of 10 nm of gold to eliminate the charging effect. Each sample was observed in three different areas with an accelerating voltage of 20 keV, in the secondary electron mode.

### Statistical Analysis

Each result is indicated as the mean value of two or three independent experiments (number of independent experiments is indicated in the caption of each figure) ± the standard derivation. The statistical analysis of all results was performed using GraphPad Prism version 5.00 for Windows (GraphPad Software, 2007), using two-way analysis of variance followed by Tukey multiple-comparisons test.

## Results

### Selection of Indium-Sensitive Mutant and Identification of Mutated Gene

Approximately 1,500 random mutants of strain B2A1Ga4, generated by Tn5 transposon mutagenesis, were tested for their ability to grow on R2A plates containing 1 mM In. One clone unable to grow in solid medium with 1 mM In, designated B2, was selected to confirm its In sensitivity in liquid medium comparatively and for detailed genetic analysis. Inverse PCR using transposon-specific primers followed by DNA sequencing of the amplified fragments identified the Tn5 insertion site in the mutant B2. Tn5 was located into an open reading frame encoding for a protein with a high degree of homology to a DedA family protein (uncharacterized membrane protein YqaA) [National Center Biotechnology Information (NCBI) Reference Sequence: WP_108473013.1], which is a highly conserved protein family poorly characterized (Doerrler et al., 2013).

**FIGURE 1:**
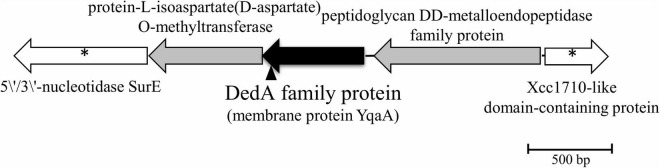
Physical map of interrupted gene from strain B2A1Ga4, identified by IPCR. Transposon insertion into *yqaA* gene is indicated by the vertical arrowhead. The sequences marked with * are sequences obtained by the draft genome sequencing of B2A1Ga4.

DNA sequencing of the inverse PCR products and analysis of the genome of *Rhodanobacter* sp. B2A1Ga4 (DDBJ/ENA/GenBank under the accession JADBJR000000000.1) showed that the mutated gene is placed between genes encoding for a peptidoglycan DD-metalloendopeptidase family protein and a protein-L-isoaspartate(D-aspartate) *O*-methyltransferase, as shown in Figure 1.

The *Rhodanobacter* sp. B2A1Ga4 genome, besides the YqaA protein (MBQ4853883) with 202 amino acids, encodes two other members of the DedA family protein, with 206 (MBQ4854938) and 170 amino acids (MBQ4855745), respectively. These two members of the DedA protein family do not have significant amino acid similarity with the YqaA protein; however, they share 25% of amino acid identity with each other.

### DedA Protein Family Sequence Alignment and Analysis

**FIGURE 2:**
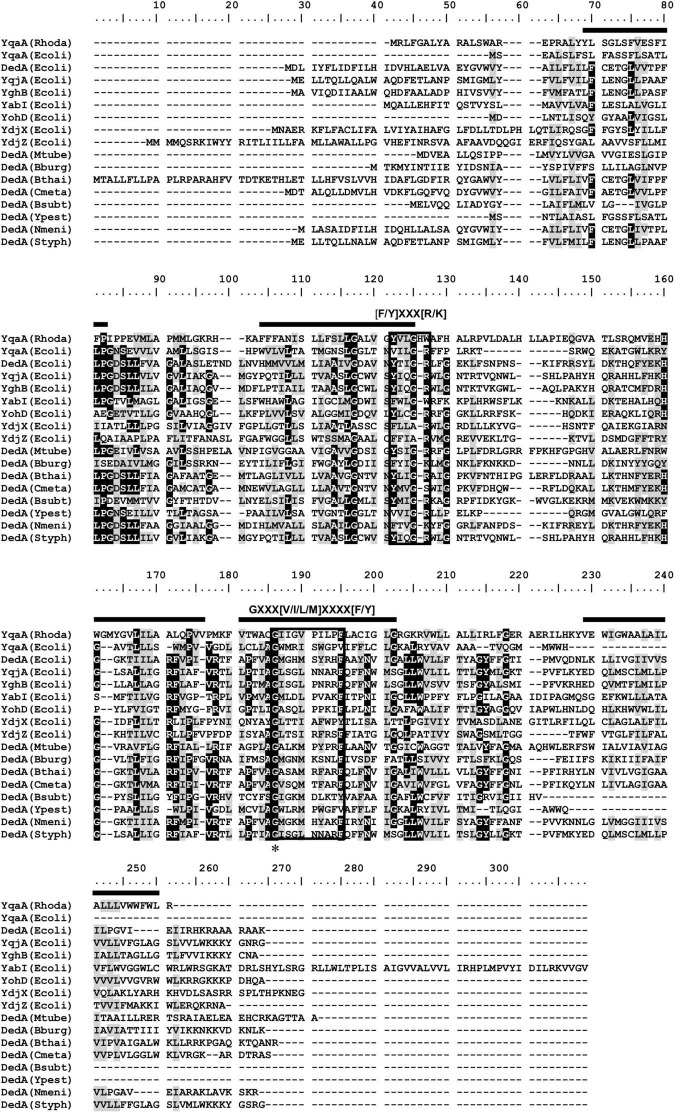
Alignment of YqaA protein of *Rhodanobacter* sp. B2A1Ga4 with homolog sequences of the DedA protein family retrieved from the NCBI database. Black boxes around the sequences show the two conserved motifs previously identified in DedA protein family (Keller and Schneider, 2013; Tábara et al., 2019). Black bars represent predicted transmembrane domains for *Rhodanobacter* sp. B2A1Ga4 obtained by the CCTOP (Constrained Consensus Topology Prediction) software (Krogh et al., 2001; Dobson et al., 2015). Asterisk indicates the position of the glycine, the fully conserved residue in all DedA proteins. Conserved (BLOSUM62) residues were shaded according to degree of conservation: black, >90%; gray, 50–90%. Rhodo, *Rhodanobacter* B2A1Ga4; Ecoli, *Escherichia coli*; Mtube, *Mycobacterium tuberculosis*; Bburg, *Borrelia burgdorferi*; Bthai, *Burkholderia thailandensis*; Cmeta, *Cupriavidus metallidurans*; Bsubt, *Bacillus subtilis*; Ypest, *Yersinia pestis*; Nmeni, *Neisseria meningitidis*; Styph, *Salmonella typhimurium*.

Alignment of YqaA protein of *Rhodanobacter* sp. B2A1Ga4 with homolog sequences of the DedA protein family showed the presence of two short motifs separated in length by approximately 50 amino acids (Figure 2), previously identified in DedA protein family (Keller and Schneider, 2013; Tábara et al., 2019). These two motifs have the consensus sequences of [F/Y]XXX[R/K] (motif 1) and GXXX[V/I/L/M]XXXX[F/Y] (motif 2), respectively, and are conserved in most of the proteins that belong to the DedA family. In YqaA protein of *Rhodanobacter* sp. B2A1Ga4 motif 1 is slightly different, having a histidine (H) and tryptophan (W) in the last two positions of motif 1. However, motif 2 is fully conserved in YqaA protein, including the glycine in the first position of motif 2, the most conserved residue in all DedA protein family. The secondary structure prediction of YqaA protein suggested that this protein is located in the cytoplasmatic membrane and contains five predicted transmembrane domains with a cytoplasmic N-terminal and an extracellular C-terminal, respectively.

### Bacterial Growths

The determination of the MIC values for both strains (the native strain and the mutant B2) was performed in liquid assays using In concentration ranging between 1 and 0.01 mM. The In MIC values were 1 and 0.5 mM for native and mutant strains, respectively. These results confirmed that gene mutation led to a loss of In resistance.

**FIGURE 3:**
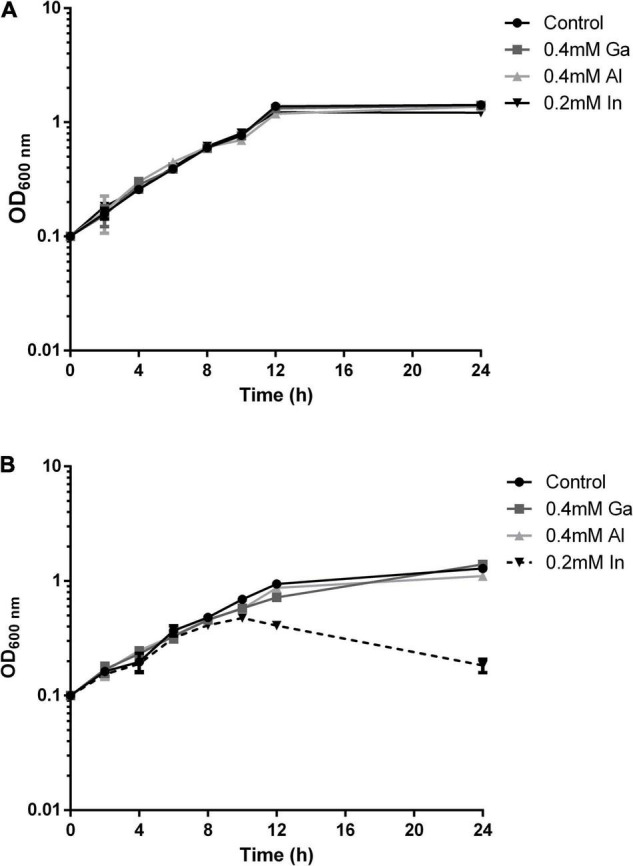
Growth curves of strain B2A1Ga4 **(A)** and mutant B2 **(B)** in different conditions: control (without metal), 0.4 mM Ga, 0.4 mM Al, and 0.2 mM In.

The growth of the native and mutant B2 strains in medium supplemented with the selected metals from the 13th group of the periodic table, In, Ga, and Al, was evaluated along with incubation time (Figure 3). The growth curves of strain B2A1Ga4 in all conditions (with or without metals) exhibited a similar profile. However, in the case of mutant B2, the bacterial growth curve was clearly different in presence of In comparatively to the control situation. This difference is particularly visible at late exponential phase (12 h of incubation). The other tested metals (Ga and Al) did not affect the growth profile of the mutant B2.

As bacterial growth might be impaired by gene mutation, native and mutant strains were tested under different conditions of temperature, presence of several antibiotics and metals, and presence of SDS. The strains exhibited similar results in the tested conditions (Table 2). Both strains showed susceptibility to chloramphenicol, gentamicin, erythromycin, nalidixic acid, rifampicin, and tetracycline and resistance to amoxicillin, ampicillin, colistin, and polymyxin B. The two strains grew at 20 and 25°C but did not grow at higher temperatures. Moreover, both strains showed the same resistance/susceptibility profile for all tested metals and SDS concentrations.

**TABLE 2:** Growth of native and mutant strains in different conditions (antibiotics, metals, and other parameters).

Antibiotics			Metals			Other parameters		
	Native	Mutant B2		Native	Mutant B2		Native	Mutant B2
AMC	R	R	0.5 mM Ni	+	+	20°C	+	+
AMP	R	R	0.5 mM Nd	+	+	25°C	+	+
C	S	S	0.5 mM As	+	+	30°C	-	-
CN	S	S	0.25 mM Y	+	+	37°C	-	-
CT	R	R	0.25 mM Sc	+	+	45°C	-	-
E	S	S	0.25 mM La	+	+	0.05% SDS	+	+
NA	S	S	0.25 mM Cu	+	+	0.2% SDS	-	-
PB	R	R	0.1 mM Zn	+	+			
RD	S	S	0.1 mM Te	-	-			
TE	S	S	0.1 mM Cr	-	-			
			0.1 mM Co	-	-			

*AMC, amoxicillin: 30 μg; AMP, ampicillin: 10 μg; C, chloramphenicol: 30 μg; CN, gentamicin: 10 μg; CT, colistin: 10 μg; E, erythromycin: 15 μg; NA, nalidixic acid: 30 μg; PB, polymixin B: 300 units; RD, rifampicin: 5 μg; TE, tetracycline: 30 μg. “+”: growth, “–”: no growth.*

The importance of the DedA family protein YqaA in conferring resistance to In in *Rhodanobacter* sp. B2A1Ga4 strain was confirmed by comparing the In MIC values and growths of the mutant B2 complemented with a functional DedA family protein (B2_p*yqaA*) with the B2 mutant control carrying the empty vector (B2_p). The In MICs for both strains, B2_p and B2_p*yqaA*, were 0.25 and 0.5 mM, respectively. These values are not the same as those obtained for native or mutant B2 strains, but this can be explained because the complemented mutant strains carry an additional plasmid with a gentamicin resistance gene that affected the bacterial behavior.

**FIGURE 4:**
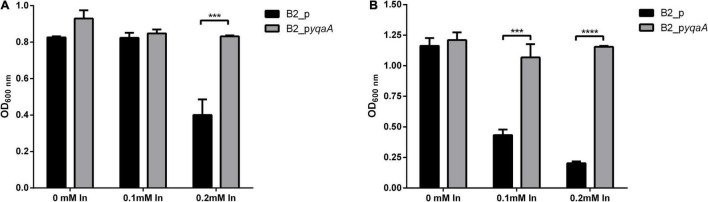
Growth of strains B2_p and B2_p*yqaA* at 16 h **(A)** and 24 h **(B)** of incubation at 25°C. Data shown are the mean values (±standard deviations) obtained from three independent experiments. ***, ****Significantly different between the two strains, *p* < 0.001 and *p* < 0.0001, respectively.

Comparing the OD_600_ measurements of the two complemented B2 strains, the growth of the control strain B2_p was significantly affected by In exposure; however, the metal did not affect the growth of strain B2_p*yqaA* (Figure 4). At 16 h of incubation time, only 0.2 mM of In had a negative effect in the growth of control cells that showed half OD_600_ of the value measured in the control condition (without In) or for strain B2_p*yqaA*. The highest differences were obtained at 24 h of incubation, showing significant differences between OD_600_ of growth of B2_p and B2_p*yqaA* strains in presence of both In concentrations tested (0.1 and 0.2 mM).

### Cellular Metabolic Activity

**FIGURE 5:**
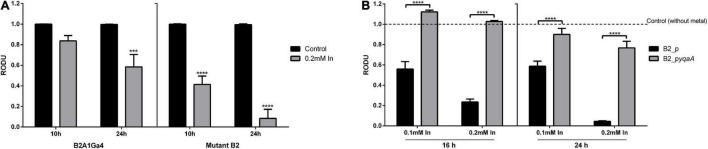
Metabolic cellular activity of the studied strains. MTT assays of strains B2A1Ga4 and B2 **(A)** MTT assays of strains B2_p and B2_p*yqaA* **(B)**. Data shown are the mean values (±standard deviations) obtained from three independent experiments. ***, ****Significantly different from the value of Control (without metal), *p* < 0.001 and *p* < 0.0001, respectively. Relative optical density units (RODU) means the ratio between the OD_550_ (absorbance at 550 nm) of the sample (test) and the OD_550_ of the control experiment.

MTT assay was used to compare the cellular metabolic activity of mutant with native strain and the *yqaA* complemented mutant (B2_p*yqaA*) with the control strain (B2_p) when exposed to In, as an indicator of cell viability or cytotoxicity (Figure 5). The native strain in presence of In showed a decrease of activity of 1.2- and 1.7-fold at 10 and 24 h of incubation, respectively, when compared to the control condition. In the case of mutant strain, In exposure resulted in a very stronger impact on cellular activity, showing a decrease of 2.4- and 12.1-fold for 10 and 24 h of incubation, respectively (Figure 5A). The results of MTT assays also showed that In exposure affected drastically the cellular activity of control strain B2_p, but had only a slight effect in strain B2_pyqaA (Figure 5B). The presence of 0.1 mM In reduced the cellular activity of strain B2_p to approximately 2-fold of the control situation at 16 and 24 h. The highest concentration of In tested (0.2 mM In) showed a much stronger impact in cellular activity of strain B2_p, with reductions of 4.3- and 22.7-fold at 16 and 24 h, respectively. B2_p*yqaA* strain showed only a minor reduction of 1.3-fold of cellular activity with 0.2 mM In at 24 h of growth. Therefore, significant differences between the metabolic activities of B2_p and B2_p*yqaA* were observed for both evaluated conditions, different In concentration and incubation time.

### Indium Accumulation

**FIGURE 6:**
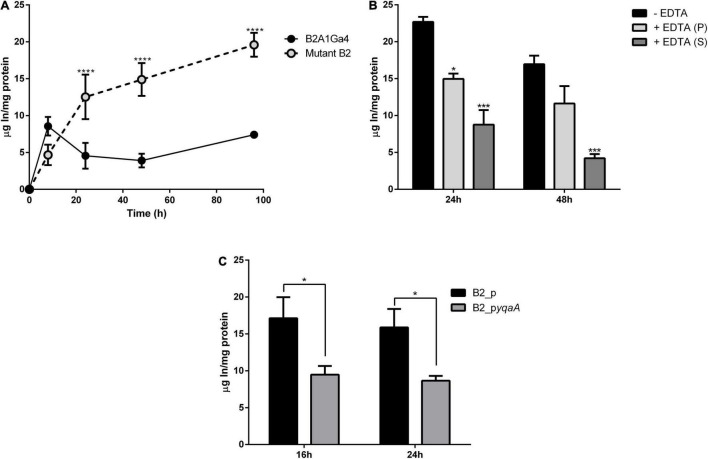
Accumulation of In by wild strain B2A1Ga4, mutant B2, and complemented strains. Total In accumulation by native and mutant B2 strains at different times: 8, 24, 48, and 96 h **(A)**. In levels in different fractions of cells collected at 48 h of growth with 0.1 mM In: without EDTA treatment—total In in cells (black), cellular fraction after EDTA treatment—intracellular In (gray), and supernatant fraction after EDTA treatment—surface In complexed (white) **(B)**. In accumulation by strains B2_p and B2_p*yqaA* (**C**). Data shown are the mean values (±standard deviations) obtained from two independent experiments. *, ***, ****Significantly different, *p* < 0.05, *p* < 0.001, and *p* < 0.0001, respectively.

The In accumulation, quantified by ICP-MS, showed that native and mutant strains have different profiles and levels of In accumulation (Figure 6A). The native strain showed the highest In accumulation values (2.80 ± 0.02 μg In/mg protein) at the exponential growth phase (8 h). For further incubation times, native cells showed lower In levels. The mutant B2 showed an increase of In amounts in the cells over the period of incubation with 4.67 ± 0.98, 12.53 ± 2.70, 14.89 ± 1.82, and 19.60 ± 1.14 μg In/mg protein at 8, 24, 48, and 96 h, respectively. These results showed that the mutant strain was able to accumulate up to 3.8-fold more In than the native strain.

The assay with EDTA was used to determine the main location of In in cells, if it was intracellular or complexed on the cellular surface. The fraction not treated with EDTA represents the total In amount in cells (intracellular and surface complexed In). After EDTA treatment, two fractions were obtained, a cellular fraction (intracellular In) and a supernatant fraction (surface complexed In). Figure 6B shows the values of In measured in these three fractions for samples collected at 48 h of growth. While the native strain showed 2.80 ± 0.02 μg In/mg protein in the total fraction, the mutant B2 showed 16.96 ± 0.82 μg In/mg protein. Mutant B2 accumulated significantly more In intracellularly than the native strain as the highest amount of In in the mutant cellular pellet corresponds to intracellular In (11.63 ± 1.67 and 1.52 ± 0.20 μg In/mg protein from mutant B2 and native strain, respectively). In the case of native cells, the highest amount of metal measured corresponds to In binding to the cellular surface (2.00 ± 0.23 μg In/mg protein). These results show that mutant strain was able to accumulate into the cells the largest fraction of In, with approximately 69% of the total In measured in cells.

In accumulation by the complemented mutant strains was evaluated to confirm the relevance of this studied DedA family protein (YqaA) in the process of In extrusion. Strain B2_p*yqaA* accumulated significantly lower amounts of In with 9.48 and 8.66 μg In/mg protein at 16 and 24 h, respectively, when compared to the control strain B2_p that showed 17.12 and 15.88 μg In/mg protein for the same incubation periods (Figure 6C). These results mean that B2_p*yqaA* cells accumulated approximately half of the In amount accumulated by the control strain.

### Membrane Potential

Impaired DedA protein function could affect the membrane potential (Δψ). Thus, Δψ was measured using the probe JC-1 red/green dye. JC-1 is a membrane permeable dye that as a monomer exhibits green fluorescence at 530 nm, and in the presence of membrane potential, it aggregates showing red fluorescence at 595 nm. Therefore, membrane potential can be expressed as the ratio between the red (595 nm) and green (530 nm) fluorescence value ratio of red to green fluorescence (Jovanovic et al., 2006; Engl et al., 2011).

**FIGURE 7:**
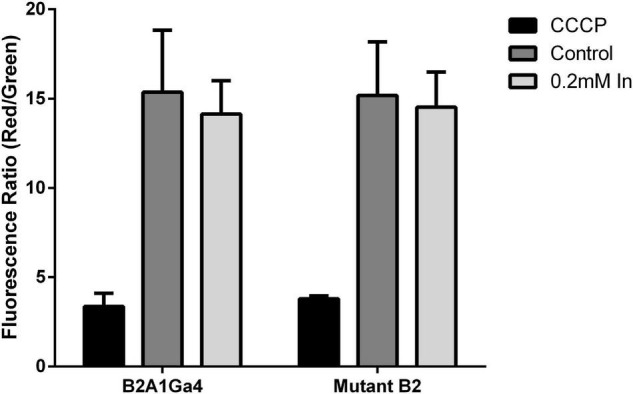
Membrane potential (Δψ) of both strains (B2A1Ga4 and mutant B2) using the probe JC-1 red/green dye, represented as fluorescence ratio red (595 nm)/green (530 nm) at different conditions: CCCP, control (without metal), and 0.2 mM In. Data shown are the mean values (±standard deviations) obtained from two independent experiments.

For both strains, native and mutant, there was no statistically significant difference in the membrane potential of cells grown in presence or absence of In (Figure 7). Moreover, fluorescence ratio values of mutant strain were similar to native strain indicating that compromised YqaA did not affect the membrane potential. The samples treated with CCCP served as a control of the experiment and showed similar fluorescence ratio.

The membrane potential was tested for both strains (B2_p and B2_p*yqaA*) with the protocol previously described (JC-1 probe). Once more, there was no significant difference in the membrane potential observed with and without the exposure to In, and the results obtained were similar for both strains (Supplementary Figure 1).

### Scanning Electron Microscopy

**FIGURE 8:**
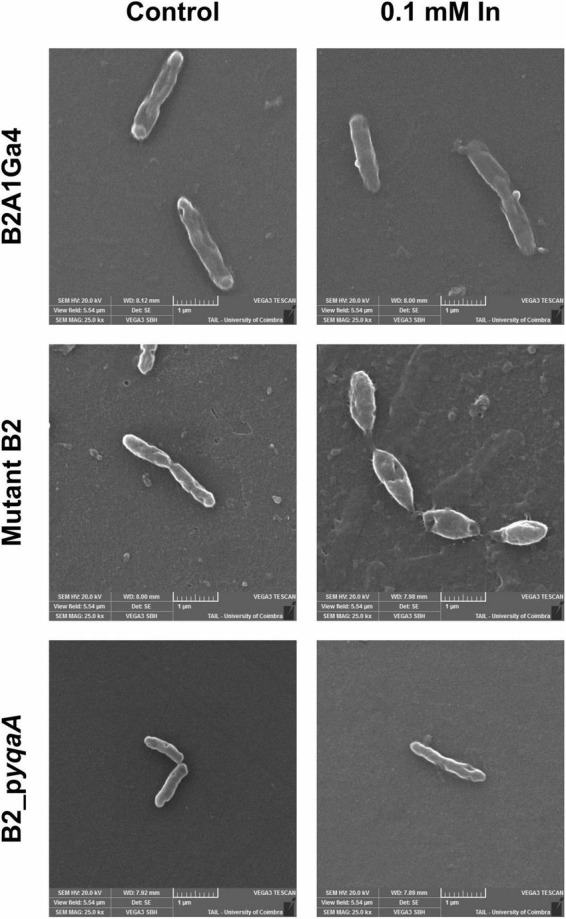
Representative SEM micrographs (magnification of 25,000×) of the different strains, B2A1Ga4, mutant B2, and B2_p*yqaA*, grown with and without 0.1 mM In.

The Figure 8 shows the SEM images of the different strains (B2A1Ga4, mutant B2 and B2_p*yqaA*) grown with and without 0.1 mM In. The presence of In does not seem to interfere with the cellular morphology of the native strain. In case of the mutant B2, the In exposure resulted in visible cellular changes, in which the cells are shorter and wider than the native strain. Moreover, the gene complementation of mutant strain reverted the cellular morphology to the original characteristics.

## Discussion

In a previous study, *Rhodanobacter* sp. B2A1Ga4 showed high resistance to In, Ga, and Al (Caldeira et al., 2021). As the resistance mechanisms to these metals are not well known, we intended to scrutinize the biological processes that mediate this bacterial ability. In our previous study, the ferrous iron transporter system FeoAB was shown to be relevant for In and Ga resistance process (Caldeira et al., 2021). As far as we know, with exception of the mentioned study and also a recent work with *S. fonticola* strains (Caldeira et al., 2020), there are no other reports about the bacterial strategies to cope with toxic compounds of In. In both reports, the resistance of *Rhodanobacter* sp. and *S. fonticola* strains involves mechanisms of detoxification of the ROS levels produced by In. The role of putative transporters in providing resistance to In by lowering the metal amount inside the microbial strains has not been investigated prior to this study. Here we show that mutation of DedA homolog YqaA affected drastically the In resistance, which indicates that this protein plays an important role in the In resistance phenotype of *Rhodanobacter* sp. B2A1Ga4 strain. DedA protein family has been particularly linked to colistin resistance in many strains (Shi et al., 2004; Tzeng et al., 2005; Weatherspoon-Griffin et al., 2011; Jana et al., 2017; Huang et al., 2019; Panta et al., 2019). These proteins were also shown to play different roles in bacteria. For instance, in *Borrelia burgdorferi* and *E. coli* the DedA family proteins are essential to cellular viability (Doerrler et al., 2013). DedA family–deficient mutants exhibit changed phenotypes such as cell division defects (Liang et al., 2010; Sikdar and Doerrler, 2010; Doerrler et al., 2013), temperature sensitivity (Thompkins et al., 2008; Doerrler et al., 2013), alkaline pH sensitivity (Price and Raivio, 2009), biocide sensitivity (Kumar and Doerrler, 2014), and compromised membrane proton-motive force (Doerrler et al., 2013; Sikdar et al., 2013; Panta et al., 2019). Although DedA is a large superfamily of membrane proteins found in eukaryote and prokaryote organisms, their molecular function and structure are largely unknown. A strictly conserved amino acid sequence is not present among all defined DedA family proteins. However, two conserved sequence motifs, [F/Y]XXX[R/K] and GXXX[V/I/L/M]XXXX[F/Y] are found in most of the proteins that are part of this family (Keller and Schneider, 2013; Tábara et al., 2019; Okawa et al., 2021). Despite minor differences, the YqaA protein sequence of *Rhodanobacter* sp. B2A1Ga4 contains these conserved sequence motifs and also the universally conserved glycine residue found in all DedA family proteins (Keller and Schneider, 2013; Tábara et al., 2019; Okawa et al., 2021). The secondary structure prediction for YqaA protein with five transmembrane domains also follows the suggested predictions for DedA protein members with five to eight TM domains (Morita et al., 2018).

In this work, we show that the growth of mutant B2–deficient in YqaA protein in the presence of In was significantly affected, as well as its cellular metabolic activity evaluated through MTT assays. This negative impact of In might be the consequence of the high accumulation of metal in the mutant cells, resulting in cellular toxicity. It is well documented that excess of metals inside the cells can be toxic, and this toxicity involves several mechanisms, such as the breaking of vital enzymatic functions, production of reactive oxygen species, disruption of ion balance, and directly affecting DNA structure, membrane lipids, and proteins (Chandrangsu et al., 2017; Igiri et al., 2018). As a result of the metal toxicity, the morphology, metabolic activity, and growth of bacteria might be affected by metal accumulation (Cervantes et al., 2001; Fashola et al., 2016). In addition, to the In sensitivity evidenced by the mutant B2, the expression of the *yqaA* gene in this mutant rescued the original bacterial phenotype with a significant decrease in levels of In accumulation in cells, increase of In resistance and increase in the metabolic activity. These findings suggest a potential role for this DedA family protein as a membrane transporter involved in the In efflux process. Thus, when this potential In extrusion system was compromised with the *yqaA* gene mutation, the mutant strain was not able to remove the excess of accumulated metal, and consequently, the high amount of In inside the cells resulted in cellular toxicity reflected in a diminished metabolic activity of mutant cells. Moreover, for the several tested biocides (antibiotics and metals), with the exception of In, the mutation did not change the bacterial resistance profile, which also supports the role of YqaA as a transporter of In from inside the cells but not being involved in the transport of other compounds.

Most published works correlate members of the DedA family with proton-dependent transporters required for PMF (proton-motive force) preservation (Doerrler et al., 2013; Keller and Schneider, 2013; Sikdar et al., 2013; Kumar and Doerrler, 2014; Panta et al., 2019). However, *yqaA* mutated and native strains grown in the presence or absence of In exhibited similar membrane potential, which indicates that the deficient YqaA protein in the mutant strain has no effect on membrane depolarization. Therefore, the high In accumulation shown by mutant strain appears to be the result of a non-functional transporter as consequence of the genetic mutation, without affecting the proton-motive force in the mutant cells. A protein evolutionary relationship analysis showed that DedA family proteins share certain structural similarities with LeuT- type transporters (Khafizov et al., 2010; Keller et al., 2014), suggesting that DedA family proteins might have a proton symporter or antiporter activity (Doerrler et al., 2013). A more recent study also proposes a potential ion-coupled transport-like function for DedA family proteins (Okawa et al., 2021).

Up to our knowledge, there is no study that relates DedA family proteins with metal membrane transporters with exception of a DedA family protein from *Ralstonia metallidurans* CH34 (renamed *Cupriavidus metallidurans* CH34) that was linked to selenite uptake (Ledgham et al., 2005).

It is reported that In enters rapidly into the bacterial cells by unclear uptake mechanisms (Aldridge and Downs, 2011), but generally bacterial strains pump this metal out of cells at the late stationary phase (Caldeira et al., 2020). The native strain B2A1Ga4 also showed this trend, extruding In in later incubation times. However, the mutant strain retains the metal for longer periods of times such as 4 days of growth, which is a very interesting feature, as bioaccumulation can contribute to a better strategy of In recovery from metal contaminated solutions.

The use of cells able to remove and concentrate metals has particular interest because biological approaches are often more environmentally friendly and economically viable (Medfu Tarekegn et al., 2020). In the case of critical metals, biobased approaches such as bioaccumulation or biosequestration associated with biomining activities have been gaining special interest, increasing the metal recovery from secondary metal sources as mine or industrial wastes (Pollmann et al., 2016). Therefore, the great In bioaccumulator resulting from this work can provide a valuable and promising technology for In remediation and recovery.

## Data Availability Statement

The datasets presented in this study can be found in online repositories. The names of the repository/repositories and accession number(s) can be found below: https://www.ncbi.nlm.nih.gov/genbank/, JADBJR000000000.1.

## Author Contributions

RB conceived and designed the experiments. JC, AC, and RB performed the experiments and wrote the manuscript. AP performed the microscopy analysis. JC, AC, PM, and RB analyzed the data. AP, PM, and RB contributed reagents, materials, and analysis tools. AC, AP, PM, and RB revised the manuscript. All authors approved the manuscript.

## Conflict of Interest

The authors declare that the research was conducted in the absence of any commercial or financial relationships that could be construed as a potential conflict of interest.

## Publisher’s Note

All claims expressed in this article are solely those of the authors and do not necessarily represent those of their affiliated organizations, or those of the publisher, the editors and the reviewers. Any product that may be evaluated in this article, or claim that may be made by its manufacturer, is not guaranteed or endorsed by the publisher.
