# Supplementary material for: A DedA Family Membrane Protein in Indium Extrusion in Rhodanobacter sp. B2A1Ga4
Source: Front Microbiol. 2021 Nov 26;12:772127. doi: 10.3389/fmicb.2021.772127 (PMC8679861; doi:10.3389/fmicb.2021.772127)
Supplement: Supplementary file 1 [file Data_Sheet_1.doc]

Supplementary Material

# A DedA Family Membrane Protein in Indium Extrusion in *Rhodanobacter* sp. B2A1Ga4

Joana B. Caldeira^1^, Ana Paula Chung^1^, Ana Paula Piedade^2^, Paula V. Morais^1^, Rita Branco^1*^

^1^University of Coimbra, Centre for Mechanical Engineering, Materials and Processes, Department of Life Sciences, Calçada Martim de Freitas, 3000-456 Coimbra, Portugal

^2^ University of Coimbra, Centre for Mechanical Engineering, Materials and Processes, Department of Mechanical Engineering, Rua Luis Reis Santos, 3030-788 Coimbra, Portugal

*** Correspondence:**Rita Branco
rbranco@uc.pt

**Supplementary Table S1.** Accession numbers for the sequences of proteins from the DedA family used in the alignment represented in Figure 2.

| **Bacteria** | **Accession numbers** | **References** |
| --- | --- | --- |
| *Rhodanobacter* sp. B2A1Ga4 | MBQ4853883 (YqaA) | This study |
| *Escherichia coli* K12 MG1655 | WP_000364335 (DedA) WP_000422149 (YqjA) WP_000268419 (YghB) WP_001148390 (YabI) WP_001296821 (YohD) WP_001300395 (YdjX) WP_000980098 (YdjZ) WP_001287454 (YqaA) | (Thompkins et al., 2008; Sikdar and Doerrler, 2010; Sikdar et al., 2013; Kumar and Doerrler, 2014; Okawa et al., 2021) |
| *Mycobacterium tuberculosis* H37Rv | WP_003413654 | (Doerrler et al., 2013) |
| *Borrelia burgdorferi* B31O1 | WP_002657683 | (Liang et al., 2010) |
| *Burkholderia thailandensis* E264 | ABC36705 | (Panta et al., 2019) |
| Cupriavidus metallidurans CH34 | WP_011517454 | (Ledgham et al., 2005) |
| *Bacillus subtilis subtilis* 168 | NP_388110 | (Okawa et al., 2021) |
| *Yersinia pestis* IP32953 | WP_002209451 | (Okawa et al., 2021) |
| *Neisseria meningitidis* MC58 | WP_002219343 | (Tzeng et al., 2005; Okawa et al., 2021) |
| *Salmonella typhimurium* 14028s | WP_000422143 | (Shi et al., 2004) |

# References

Doerrler, W. T., Sikdar, R., Kumar, S., and Boughner, L. A. (2013). New functions for the ancient DedA membrane protein family. *J. Bacteriol.* 195, 3–11. doi:10.1128/JB.01006-12.

Kumar, S., and Doerrler, W. T. (2014). Members of the conserved DedA family are likely membrane transporters and are required for drug resistance in *Escherichia coli*. *Antimicrob. Agents Chemother.* 58, 923–930. doi:10.1128/AAC.02238-13.

Ledgham, F., Quest, B., Vallaeys, T., Mergeay, M., and Covès, J. (2005). A probable link between the DedA protein and resistance to selenite. *Res. Microbiol.* 156, 367–374. doi:10.1016/j.resmic.2004.11.003.

Liang, F. T., Xu, Q., Sikdar, R., Xiao, Y., Cox, J. S., and Doerrler, W. T. (2010). BB0250 of *Borrelia burgdorferi* is a conserved and essential inner membrane protein required for cell division. *J. Bacteriol.* 192, 6105–6115. doi:10.1128/JB.00571-10.

Okawa, F., Hama, Y., Zhang, S., Morishita, H., Yamamoto, H., Levine, T. P., et al. (2021). Evolution and insights into the structure and function of the DedA superfamily containing TMEM41B and VMP1. *J. Cell Sci.* 134, jcs.255877. doi:10.1242/jcs.255877.

Panta, P. R., Kumar, S., Stafford, C. F., Billiot, C. E., Douglass, M. V., Herrera, C. M., et al. (2019). A DedA family membrane protein is required for *Burkholderia thailandensis* colistin resistance. *Front. Microbiol.* 10, 2532. doi:10.3389/fmicb.2019.02532.

Shi, Y., Cromie, M. J., Hsu, F. F., Turk, J., and Groisman, E. A. (2004). PhoP-regulated *Salmonella* resistance to the antimicrobial peptides magainin 2 and polymyxin B. *Mol. Microbiol.* 53, 229–241. doi:10.1111/j.1365-2958.2004.04107.x.

Sikdar, R., and Doerrler, W. T. (2010). Inefficient Tat-dependent export of periplasmic amidases in an *Escherichia coli* strain with mutations in two DedA family genes. *J. Bacteriol.* 192, 807–818. doi:10.1128/JB.00716-09.

Sikdar, R., Simmons, A. R., and Doerrler, W. T. (2013). Multiple envelope stress response pathways are activated in an *Escherichia coli* strain with mutations in two members of the DedA membrane protein family. *J. Bacteriol.* 195, 12–24. doi:10.1128/JB.00762-12.

Thompkins, K., Chattopadhyay, B., Xiao, Y., Henk, M. C., and Doerrler, W. T. (2008). Temperature sensitivity and cell division defects in an *Escherichia coli* strain with mutations in *yghB* and *yqjA*, encoding related and conserved inner membrane proteins. *J. Bacteriol.* 190, 4489–4500. doi:10.1128/JB.00414-08.

Tzeng, Y. L., Ambrose, K. D., Zughaier, S., Zhou, X., Miller, Y. K., Shafer, W. M., et al. (2005). Cationic antimicrobial peptide resistance in *Neisseria meningitidis*. *J. Bacteriol.* 187, 5387–5396. doi:10.1128/JB.187.15.5387-5396.2005.
