# Supplementary material for: A DedA Family Membrane Protein in Indium Extrusion in Rhodanobacter sp. B2A1Ga4
Source: Front Microbiol. 2021 Nov 26;12:772127. doi: 10.3389/fmicb.2021.772127 (PMC8679861; doi:10.3389/fmicb.2021.772127)
Supplement: Supplementary file 2 [file Image_1.PDF]

Supplementary Material

A DedA Family Membrane Protein in Indium Extrusion in *Rhodanobacter* sp. B2A1Ga4

Joana B. Caldeira[1], Ana Paula Chung[1], Ana Paula Piedade[2], Paula V. Morais[1], Rita Branco[1*]

[1]University of Coimbra, Centre for Mechanical Engineering, Materials and Processes, Department of Life Sciences, Calçada Martim de Freitas, 3000-456 Coimbra, Portugal

[2] University of Coimbra, Centre for Mechanical Engineering, Materials and Processes, Department of Mechanical Engineering, Rua Luis Reis Santos, 3030-788 Coimbra, Portugal

*** Correspondence:**
Rita Branco
rbranco@uc.pt

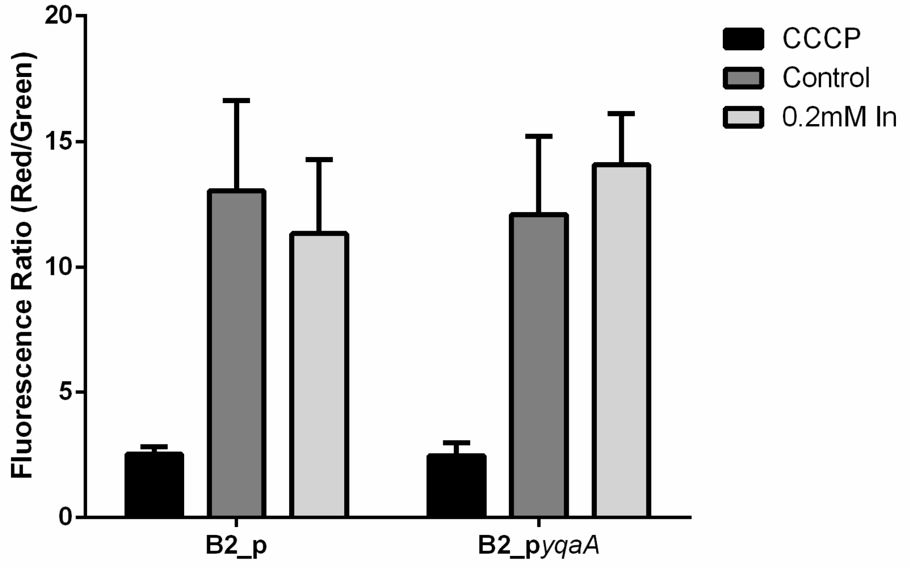

Supplementary Figure 1. Membrane potential (Δψ) of both strains (B2_p and B2_p*yqaA*) using the probe JC-1 red/green dye, represented as fluorescence ratio red (595 nm)/green (530 nm) at different conditions: CCCP, control (without metal) and 0.2 mM In. Data shown are the mean values (± standard deviations) obtained from two independent experiments.
